# Transcriptional analysis for the difference in carotenoids accumulation in flesh and peel of white-fleshed loquat fruit

**DOI:** 10.1371/journal.pone.0233631

**Published:** 2020-06-26

**Authors:** Shicheng Zou, Muhammad Qasim Shahid, Chongbin Zhao, Man Wang, Yunlu Bai, Yehua He, Shunquan Lin, Xianghui Yang

**Affiliations:** State Key Laboratory for Conservation and Utilization of Subtropical Agro-bioresources, South China Agricultural University, Tianhe District, Guangzhou, China; Institute for Horticultural Plants, China Agricultural University, CHINA

## Abstract

Loquat (*Eriobotrya japonica* Lindl.) is divided into yellow- and white-fleshed based on the difference in fruit color, and the variations in carotenoids accumulation are considered as the main reasons for this difference. Using RNA-seq technology, a transcriptome analysis was carried out on the flesh and peel of ‘Baiyu’ fruit during four different fruit development stages. A total of 172.53 Gb clean reads with an average of 6.33 Gb reads were detected for each library, and the percentage of Q30 was higher than 90.84%. We identified 16 carotenogenic and 13 plastid-lipid-associated protein (*PAP*) genes through RNA-seq. Of these, five carotenogenic and four *PAP* related genes exhibited remarkable differences in the expression patterns. Carotenoids biosynthetic genes, including *DXS*, *PSY1* and *VDE* displayed higher expression levels in peel than that in the flesh. However, carotenoids decomposition gene, such as *NCDE1*, exhibited higher expression in flesh than that in the peel. Notably, all differentially expressed *PAP* genes showed higher expression levels in peel than flesh. We inferred that the differential accumulation of carotenoids in flesh and peel of 'Baiyu' is caused by the up- or down-regulation of the carotenogenic and *PAP* related genes. The functional analysis of these important genes will provide valuable information about underlying molecular mechanism of carotenoids accumulation in loquat.

## Introduction

Fruit color formation depends on the pigment accumulation, and the types and contents of pigments determine the shades and color of fruit. The carotenoids are the major pigment in many fruits, which not only cause a difference in fruit color, but also a synthetic precursor of photosynthetic auxiliary pigment, ABA and some aromatic substances. Carotenoids biosynthesis and accumulation have attracted great attention because of the physiological role and human health-care functions [1, 2]. After the identification of carotenoids biosynthesis pathway in plants, the expression and cloning of carotenogenic genes in different plants have become a research hotspot [3–6]. However, recent studies have shown that the carotenoids accumulation might be not related to carotenogenic gene expression. The cauliflower with *Or* gene accumulated high level of *β*-carotene in flower buds and turned into orange color, but this increase in *β*-carotene was not due to the high capacity of carotenoids biosynthesis [7–9]. Chromoplast-specific carotenoid-associated synthase (CHRC) belongs to *PAP* (plastid-lipid-associated protein) family, which can promote the accumulation of carotenoids in chromoplast [10]. The expression of *CHRC* in tomato plays an important role in plastid development and carotenoids accumulation [11]. The accumulation of carotenoids in red sweet orange mutants is related to the development of chromoplastid, and there is no involvement of key genes of carotenoids metabolic pathway [12].

Loquat, *Eriobotrya japonica* Lindl., originated in China and has been cultivated for more than 2000 years in China. Loquat fruit can be grouped into two main categories based on its flesh color, white-fleshed and yellow-fleshed, which is caused by the difference in carotenoids accumulation in loquat fruit [13–15]. After unremitting efforts, many loquat varieties with white-fleshed and yellow-fleshed have been developed. Generally, the peel of white- and yellow-fleshed loquat cultivars can accumulate carotenoids normally, while white-fleshed loquat cultivars can’t accumulate or accumulate little carotenoids compared to yellow-fleshed loquat cultivars [2]. Several previous studies have demonstrated the reasons for difference in carotenoids accumulation in loquat. For example, plastids and plastid lipid-associated protein (PAP) expression patterns in two loquat cultivars (‘Luoyangqing’ and ‘Baisha’) during different ripening stages displayed differences in the structure and quantity of chromoplasts in peel and flesh, and also in the expression levels of *PAP* [16]. They speculated that the failure of ‘Baisha’ flesh to make chromoplasts was the most likely reason for the less accumulation of carotenoids. The inability of carotenoids accumulation in white-flesh loquat may associate with the non-functional mutant, *EjPSY2A* [17]. However, another study reported that the expression patterns of *BCH*, *CYCB* and *PSY* jointly control the carotenoids accumulation between ‘Zaozhong No. 6’ and ‘Baiyu’ [18]. The up-regulation of *ZDS*, *CYCB*, *PSY1* and *BCH* genes and down-regulation of *CRTISO*, *LCYE*, *ECH* and *VDE* genes were the main reasons for differential accumulation of carotenoids between flesh and peel of yellow-flesh loquat cultivar [19]. Three loquat cultivars were used to detect differences between carotenoids accumulation during a single fruit development stage [20], which is hard to explain the carotenoid accumulation in a fruit. All the previous studies on carotenoids accumulation of loquat fruit mainly focused on different cultivars (yellow- and white-fleshed cultivars) or single cultivar (yellow-fleshed cultivar), however, the genetic backgrounds of different types of loquat cultivars were quite different. In addition, the yellow-fleshed cultivars, such as ‘Obusa’, can accumulate carotenoids normally in their flesh and peel. These factors may cause differences in the carotenoids accumulation.

Here, white-fleshed cv. ‘Baiyu’ was selected as an experimental material. This cultivar is characterized by pure white flesh and yellow peel [21], and there is a significant difference in carotenoids accumulation between flesh and peel. Our previous study also indicated that the content of carotenoids in peel increased from 26.76 μg·g^-1^FW and peaked at 35.30 μg·g^-1^FW; while in the flesh it decreased slightly, from 3.39 μg·g^-1^FWto 1.72μg·g^-1^FW as the ‘Baiyu’ fruit ripened [22]. Until now, there is no report on difference in carotenoids accumulation between flesh and peel of white-fleshed loquat fruit. Therefore, it is an excellent material to study the differences in carotenoids accumulation. We analysed the transcriptome of flesh and peel at four different fruit development stages to detect differentially expressed genes (DEGs) associated with carotenoids accumulation. Consequently, the physiological investigations about the flesh and peel at different fruit development stages will contribute to our understanding about the difference in carotenoids accumulation in different loquat tissues. Moreover, RNA-Seq will be helpful to identify DEGs associated with loquat flesh color.

## Materials and methods

### Fruit material and experimental design

**Fig 1:**
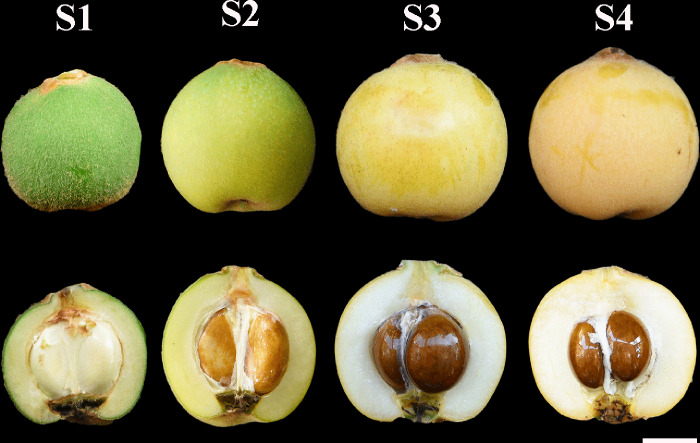
Four different development stages of ‘Baiyu’ fruit. S1: hard mature green; S2: breaker; S3: half ripen; S4: full ripen; bar 1cm.

The flesh and peel of cv. ‘Baiyu’ at four different stages (S1-S4) were selected in this study (Fig 1). For each stage, 18 uniform fruits were selected based on size and color, and fruits were divided into three parts (6 fruits per section), representing three biological replications. For RNA extraction and sequencing, fresh samples of both peel and flesh were frozen in liquid nitrogen and kept at -80°C until RNA extraction. ‘Baiyu’ is a famous cultivar, and planted at the fruit orchard of South China Agricultural University (SCAU), Guangzhou, China. The day-night length period is about 12–12 h in March, and the average temperature is ranged from 14 to 22°C during March. Guangzhou climate is subtropical, with relatively humid weather throughout the year, especially during March/April [20].

### RNA extraction, cDNA library preparation and illumina sequencing

Total RNA was extracted from three bulked biological replicates of loquat fruits at each development stage using Quick RNA isolation Kit (Bioteke Corporation, Beijing, China). RNA concentration was checked using NanoDrop 2000 (Thermo), and RNA integrity was determined using the RNA Nano 6000 Assay Kit of the Agilent Bioanalyzer 2100 system (Agilent Technologies, CA, USA).

A total amount of 1ug RNA per sample was used as input material for the RNA sample preparations. Sequencing libraries were prepared using NEBNext UltraTM RNA Library Prep Kit for Illumina (NEB, USA) following manufacturer’s recommendations as described previously [23]. In short, mRNA was filtered from total RNA using poly-T oligo-attached magnetic beads. Fragmentation was done using divalent cations under elevated temperature in NEBNext First Strand Synthesis Reaction Buffer (5X). First strand cDNA was produced using random hexamer primer and M-MuLV Reverse Transcriptase. Second strand cDNA was synthesized using DNA Polymerase I and RNase H. After adenylation of 3’ends of DNA fragments, NEBNext Adaptor with hairpin loop structure was ligated to prepare for hybridization. The library fragments were purified with AMPure XP system (Beckman Coulter, Beverly, USA) to pick out cDNA fragments, especially of 240 bp in length. After that, 3 μL USER Enzyme (NEB, USA) was used with selected cDNA, and adaptor-ligated at 37°C for 15 min followed by 5 min at 95°C. Then PCR was executed with Phusion High-Fidelity DNA polymerase, Index (X) Primer and Universal PCR primers. Finally, PCR products were filtered (AMPure XP system) and library quality was evaluated on the Agilent Bioanalyzer 2100 system.

A cBot Cluster Generation System was used for clustering of the index-coded samples by using TruSeq PE Cluster Kit v4-cBot-HS (Illumina) according to the manufacturer’s protocol. After clustering, the libraries were sequenced on an Illumina platform and paired-end reads were generated.

### Data filtration, comparative analysis and gene functional annotation

The high quality or clean reads were obtained as described previously [24]. First, we obtained clean reads by removing reads having reads containing ploy-N, adapter, and low quality reads from raw data. Meanwhile, sequence duplication level and Q30 contents of the clean data were estimated. All the downstream analyses were calculated based on clean data with high quality.

Then, the low-quality sequence reads and adaptor sequences were detached from the data sets. Raw sequences were transformed into clean reads after data processing. These clean reads were then mapped onto the reference genome. Only reads with one mismatch or a perfect match were further examined and annotated based on the loquat reference genome (unpublished data). Hisat2 tools software was used to map onto the reference genome.

Gene functional annotation was done based on the following databases: KOG/COG (Clusters of Orthologous Groups of proteins); Nr (NCBI non-redundant protein sequences); KO (KEGG Ortholog database); Nt (NCBI non-redundant nucleotide sequences); Pfam (Protein family); Swiss-Prot (A manually annotated and reviewed protein sequence database); and gene ontology (GO) enrichment analysis.

### Digital gene expression analysis

The reads per kb per million fragments (RPKM) method was used to estimate the gene expression levels [25]. Benjamini-Hochberg’s method was used for gene expression difference analysis and to correct the *p* values [26]. To determine the significance of gene expression differences between samples, FDRɤ0.01 and the absolute log_2_ ratio ≥2 was used as threshold.

### Validation of expression levels by real-time qRT-PCR

We designed specific primers by Primer Premier 5 software to validate the expression patterns of fifteen color related genes by qRT-PCR. The qRT-PCR was performed with a Light CyclerR 480 system (Roche, United States). The amplification system consisted of 5μL iTaqTM universal SYBR^®^ Green Supermix (2*), 0.5μL of 10 μmol/L upstream primer, 0.5 μL of 10 μmol/L downstream primer, 1 μL template, and sterile distilled water to a total volume of 10 μL. The amplification program was 95°C for 30 s, with 40 cycles of 95°C for 10 s, 60°C for 20 s, and 72°C for 15s. The relative quantitative analysis was done by the 2^–ΔΔCT^ method, and *EjACT* (AB710173.1) was used as reference gene [27]. To ensure reproducibility and reliability of data, three biological replicates were used for each sample. Analysis of variance (ANOVA) and Duncan’s multiple range test were executed with SPSS Version 16.0 (Chicago, IL, USA) and the significance level was set as *P* < 0.05.

## Results

### Illumina paired-end sequencing and functional annotation

For RNA-Seq analysis, twenty-four cDNA libraries were prepared from fruits of cv. ‘Baiyu’ at four different development stages. After removing low-quality reads and trimming adapter sequences, we obtained 172.53 Gb clean reads with an average 6.33 Gb clean reads for each library. The average GC contents of S1, S2, S3 and S4 were 47.14%, 47.60%, 47.27% and 47.51%, respectively. The high quality reads were mapped onto the loquat reference genome and about 89.53% to 92.42% were uniquely mapped in each sample, and covered 90.0, 90.0, 91.0, and 91.0% of reference genome at 20× coverage depth. These results showed that sequencing was of high quality and our results are reliable.

**Fig 2:**
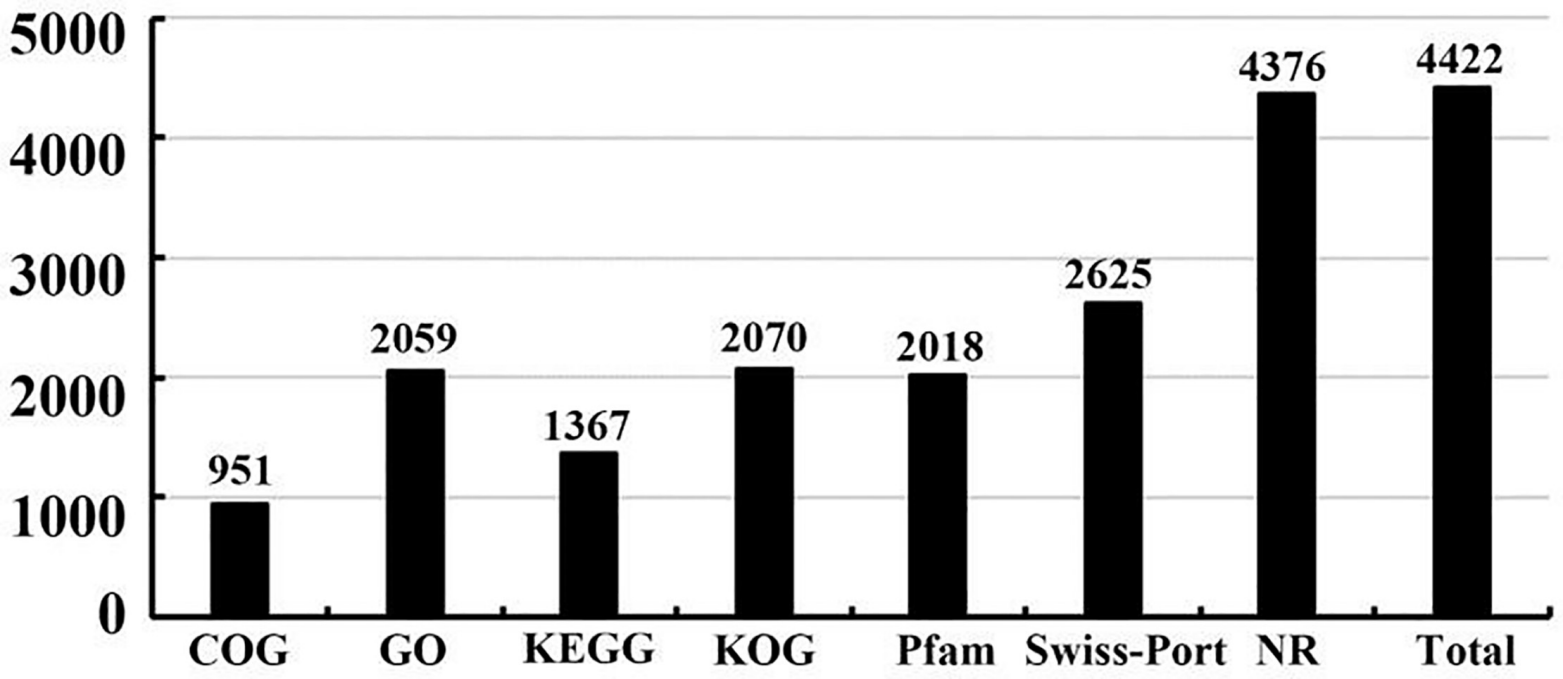
Unigenes numbers annotated in the seven public databases.

Sequence similarity searches were performed in seven public databases to validate and annotate the assembled unigenes. Of 5,733 unigenes, 951 (16.59%) showed a significant resemblance to known proteins in the COG database, 2,059 (35.91%) in the GO database, 1,367 (23.84%) in the KEGG, 2,070 (36.11%) in the KOG database, 2,018 (35.20%) in the PFAM database, 4,376 (76.33%) in the NR database and 2,626 (45.80%) in the “Swiss-Prot database” (Fig 2). In total, 4,422 (77.13%) unigenes were found to be similar with known proteins in at least one of these seven databases.

### Identification of differentially expressed genes (DEGs) and their functional annotation

**Fig 3:**
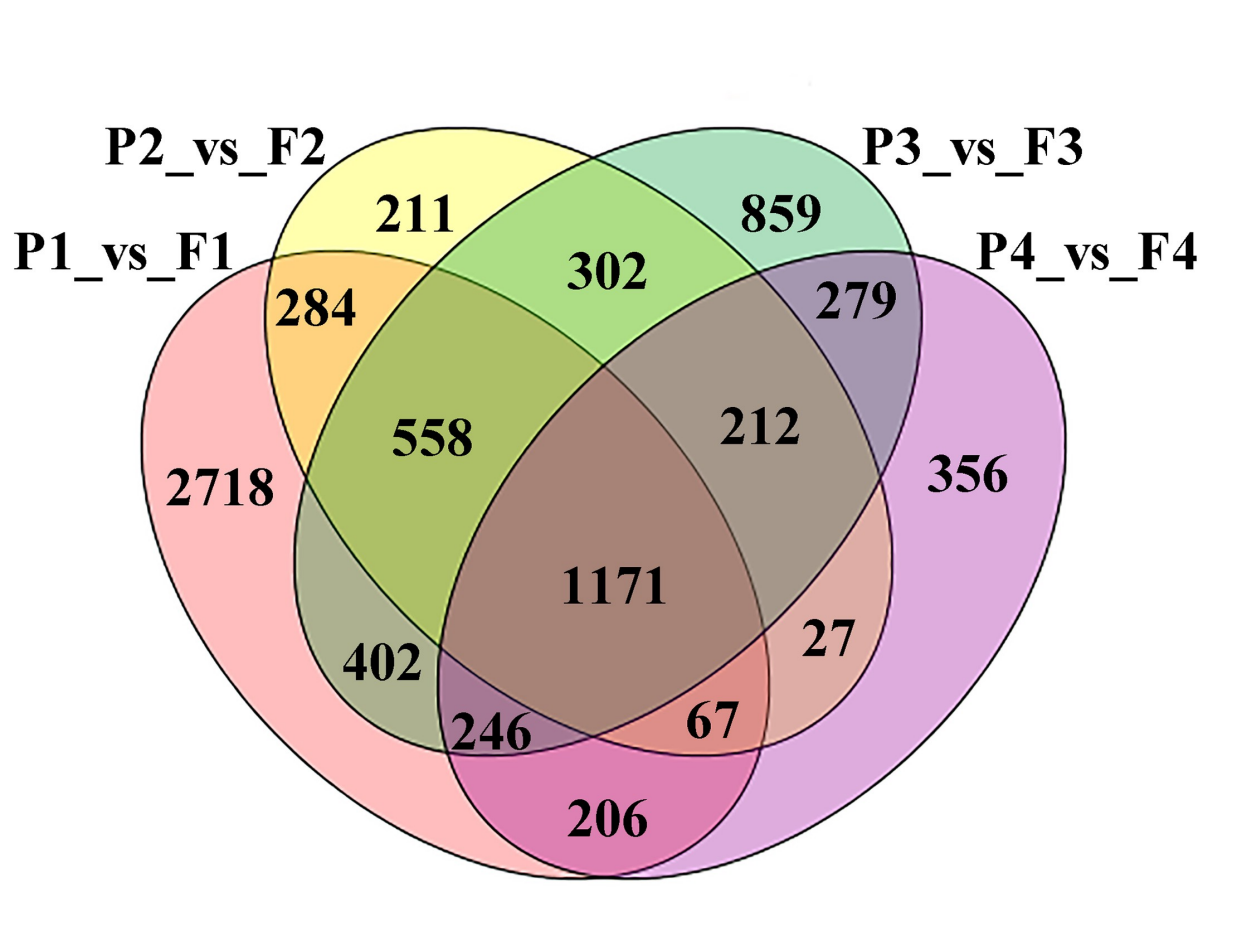
Differentially expressed genes (DEGs) detected between different comparisons in loquat. P and F represent peel and flesh, while 1,2,3 and 4 indicate four development stages, i.e. S1, S2, S3 and S4.

To detect DEGs related to flesh and peel at different fruit development stages, R package was used to identify DEGs from different samples [28]. In total, 7,898 DEGs were obtained by four different comparisons (i.e., comparison between flesh and peel at four development stages, S1-S4) (Fig 3). A total of 5,652 genes showed differential expression patterns between peel and flesh at S1, while 3,444 and 2,208 genes displayed up-regulation and down-regulation, respectively. In total, 2,832 DEGs were detected between peel and flesh at S2, including 1,828 upregulated genes and 1,004 down-regulated genes. At S3, 4,029 DEGs were detected, and 2,652 exhibited up-regulation and 1,377 down-regulation, while 2,564 DEGs were identified between peel and flesh at S4. Of these, 914 and 1,650 displayed down-regulation and up-regulation, respectively (Table 1).

**Table 1:** Analysis of differentially expressed genes (DEGs) between flesh and peel during different development stages.

DEG Set Flesh_vs_Peel	Number of DEGs	up-regulated	down-regulated
S1	5,652	3,444	2,208
S2	2,832	1,828	1,004
S3	4,029	2,652	1,377
S4	2,564	1,650	914

S1-S4 represent development stages.

**Fig 4:**
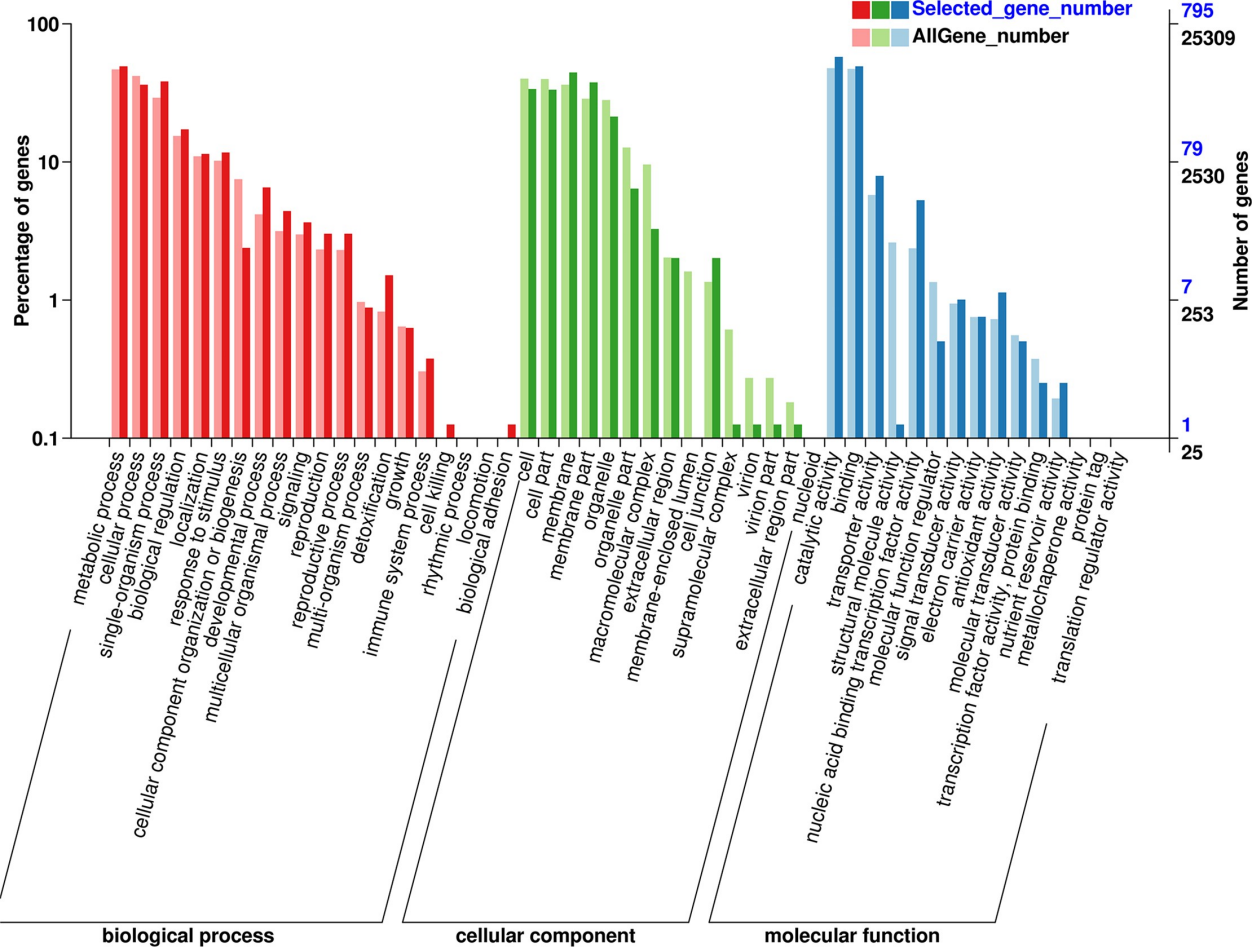
Gene Ontology (GO) classifications of the assembled unigenes. The results are summarized in three categories i.e. cellular component, molecular function and biological process category.

Gene ontology enrichment analysis of the 25,309 unigenes exhibited association with known proteins of 50 GO classes containing 124,547 functional terms. Among them, the majority of GO terms were identified in the cellular component category (51,017; 40.96%) followed by biological process category (45,523; 36.55%), and molecular function category (28,007; 22.49%) (Fig 4).

### Investigation of differentially expressed genes related with carotenoid accumulation between flesh and peel of loquat

**Fig 5:**
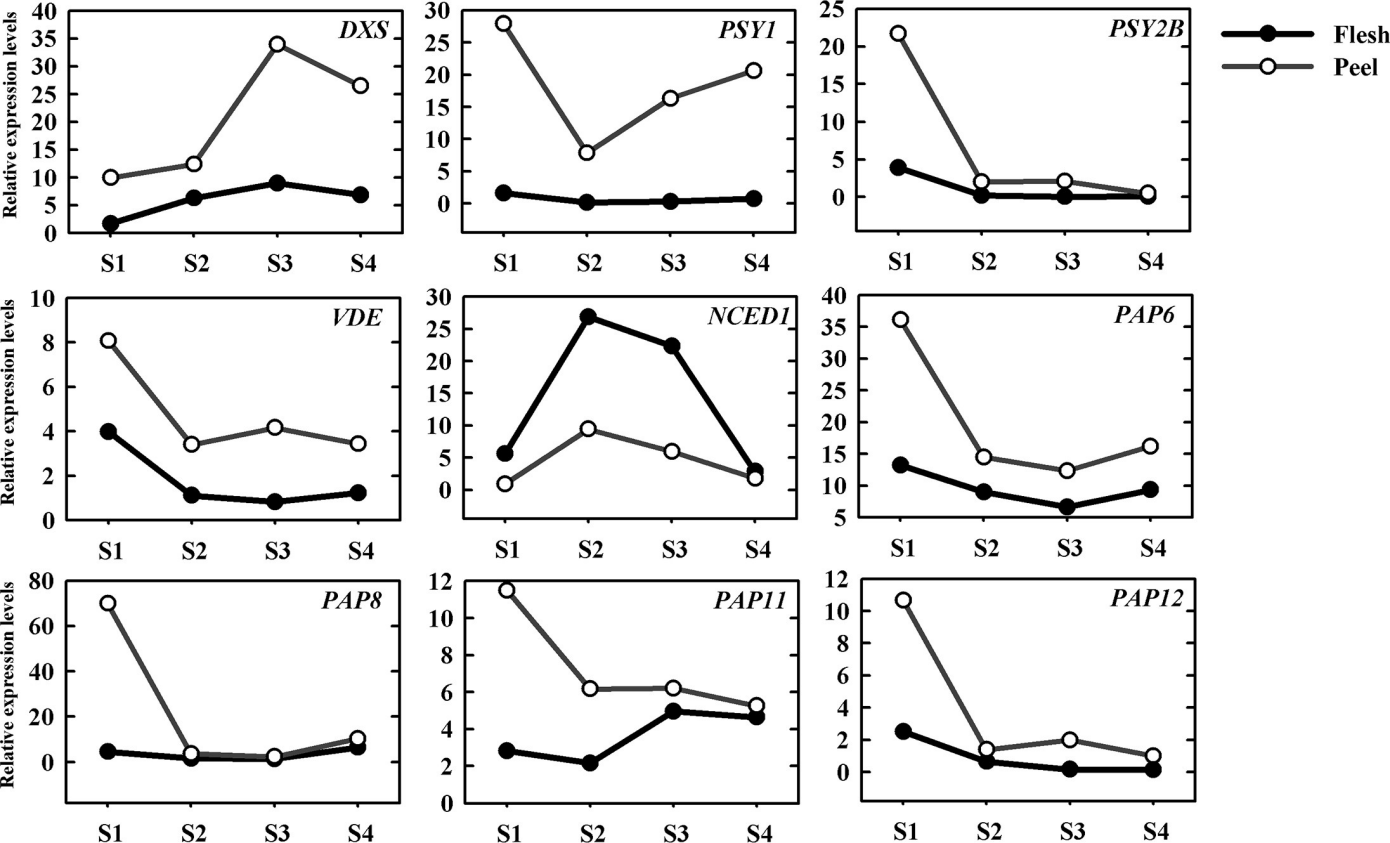
Expression patterns of carotenoid biosynthesis and *PAP* genes in flesh and peel during fruit development stages.

From RNA-seq data, we identified 16 carotenogenic genes and 13 *PAP* genes, among them, five carotenogenic genes, including *PSY1*, *PSY2B*, *DXS*, *VDE*, and *NCED1* were found to be differentially expressed between flesh and peel, and four *PAP* genes, including *PAP6*, *PAP8*, *PAP11*, and *PAP12* displayed differential expression patterns between flesh and peel (Fig 5).

*PSY1* showed a similar expression profile (first decreased and then increased”, and peaked at S1 stage) during ripening in flesh and peel. However, the *PSY1* expression level was much higher in peel than in flesh. The expression levels of *PSY2B* decreased continuously both in flesh and peel during ripening, but the expression of *PSY2B* in flesh was almost undetectable at the S3 and S4 stages. The expression of *DXS* in flesh and peel has similar expression patterns, continuously increased, and reach the highest level at S3 stage and then slightly decreased, but low expression level was detected in flesh. The expression level of *VDE* was the highest at S1, and then decreased during ripening, however, the expression of *VDE* was higher in peel than in flesh. The expression pattern of *NCDE1* in flesh and peel was similar, while the expression level of *NCDE1* in flesh was higher than in peel.

*PAP* gene is related to the differentiation and development of plastids. In our study, four differentially expressed *PAP* genes were detected during fruit development stages. *PAP6* and *PAP8* showed similar expression patterns in flesh and peel. The expression levels of *PAP6* and *PAP8* decreased with fruit development and then increased at S4, but peaked at slightly different stages (S1 stage for *PAP6* in flesh and peel, S4 stage for *PAP8* in flesh and S1 stage for *PAP8* in the peel). Overall, their expression levels were higher in peel than that in flesh. *PAP8* showed a major decrease at S2 stage and remained low thereafter, and were slightly higher in peel than in flesh. The expression patterns of the *PAP11* gene increased slightly in flesh and reached the highest at S3 stage during ripening, however it’s expression was low in peel. The expression level of *PAP12* decreased during ripening both in flesh and peel, but to a lower level in flesh than peel.

### qRT-PCR validation of the transcriptomic data

**Fig 6:**
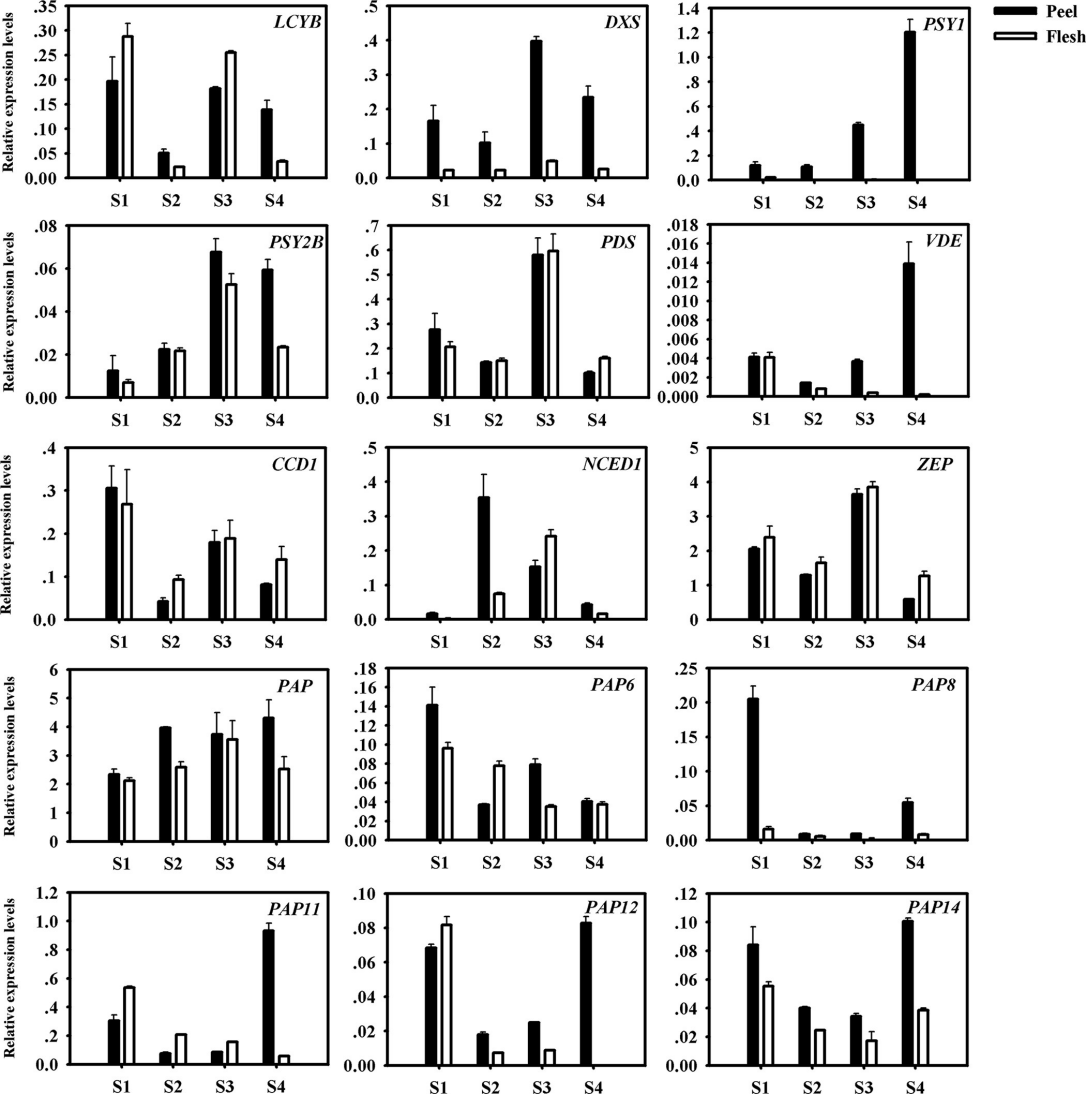
Expression levels of representative genes in flesh and peel at S1-S4 stages validated by qRT-PCR.

To validate the key RNA-seq results, we randomly selected 15 DEGs (9 carotenogenic genes and 6 *PAP* genes) and analyzed their expression levels in flesh and peel of fruit during S1-S4 development stages using qRT-PCR. The expression patterns of these genes were very similar to the RNA-seq results, with correlation coefficients (R^2^) > 0 .67 (Fig 6). The qRT-PCR results showed that expression levels of differentially expressed genes were consistent with RNA-seq data, which confirmed the reliability of our data.

## Discussion

It is well known that the carotenoids contents in plant tissues, especially flowers and fruits, varies widely within the same species. In loquat, difference in flesh color, from white to orange-yellow, are caused by different levels of carotenoids. Even different tissues have diverse carotenoids composition and content, which leads to color differences, such as cv. Baiyu, the main carotenoids in the flesh are lutein, but *β*-carotene is the main carotenoid in the peel [18]. Several previous studies have focused on the difference in carotenoids accumulation in plants, and revealed that *PSY* may play an important role for accumulation of carotenoids in many plants [29–31]. In loquat, Fu et al [17] revealed that *EjPSY1* is responsible for carotenoids synthesis in the peel but not in the flesh, whereas *EjPSY2A* is responsible for carotenoids accumulation in the flesh of ripening fruit. In the present study, the expression level of *PSY1* has the same profile in flesh and peel, but its expression level in peel was much higher than that in flesh. At the same time, the expression level of *PSY2B* declined continuously in flesh and peel during ripening and did not express in flesh at S3 and S4 stages, which was consistent with the previous study [16]. However, it is worth to mention that *PSY2A* highly expressed in flesh and peel during fruit ripening, and the difference was non-significant. This result was different from the previous studies, and need further investigations.

In addition, both *DXS* and *VDE* are carotenoids biosynthetic genes, and a previous study indicated that the over-expression of the *DXS* gene can increase the phytoene and *β*-carotene in the strain and tuber of tomato [32, 33]. The expression level of *VDE* might be related to the synthesis of violaxanthin and zeaxanthin, but the proportion of violaxanthin and zeaxanthin was low in the total contents of carotenoids [16]. Our study showed that the expression levels of *DXS* and *VDE* were significantly higher in peel than that in the flesh, which illustrated the reason for higher accumulation of carotenoids in peel of ‘Baiyu’ fruit than flesh. *NCDE1* belongs to carotenoids decomposition gene, and the contents of lycopene and *β*-carotene in tomato mature fruit can be increased by specifically inhibiting the expression of *NCED* by RNAi [34]. In apricot, high expression level of *NCDE* cause reduction in *β*-carotene content in a white-fleshed cultivar ‘Dabaixing’ [35]. Our results also confirmed this phenomenon and showed that *NCDE1* expression level in flesh was significantly higher than that in peel, which means more carotenoids retained in the peel compared to flesh. The differential expressions of these genes may explain the possible mechanism of carotenoids accumulation in flesh and peel of ‘Baiyu’.

In higher plants, the complete pathway of carotenoids biosynthesis takes place within the plastid, and carotenoids accumulation depends on the level of gene expression and enzyme activity and also on the size and number of plastids [36]. The plastid lipid-associated protein gene (*PAP*) has been reported to be involved in carotenoids sequestration in plastids [37]. Our previous study showed the normal development of chloroplast in peel at S1 stage, which gradually disappeared and replaced by the yellow-orange colored chromoplast in 'Baiyu' fruit [38]. On the contrary, many small abnormal chloroplasts were detected in the flesh at S1 stage, and the chloroplast disappears, but the formation of the chromoplast is not observed in the flesh during fruit ripening [38]. Here, thirteen *PAP* genes were identified, among them, *PAP6*, *PAP8*, *PAP11*, and *PAP12* were differentially expressed in flesh and peel. The expression levels of *PAP6*, *PAP8*, *PAP11*, and *PAP12* in peel reached the highest level at S1 stage, especially the expression level of *PAP8* in peel was about 10 times higher than that in flesh. The main plastids in flesh and peel of ‘Baiyu’ are chloroplasts at this stage, and we believed that the high expression level of these genes at this stage might be related to the formation of chloroplasts. Chloroplasts can be transformed into chromoplasts [39], which are the main sites of carotenoids synthesis and accumulation, and eventually leads to the difference in carotenoids accumulation in the flesh and peel of ‘Baiyu’. These results were consistent with the previous studies, who also detected high expression levels of plastid related genes [19, 35, 40]. Similarly, some other studies reported that the increase in carotenoids contents of the *hp1-3* tomato mutant was due to the increase in quantity and size of plastid, which provide enough space for carotenoids biosynthesis and accumulation [41–43].

In conclusion, our results revealed that the differential accumulation of carotenoids in flesh and peel of 'Baiyu' was caused by the up-regulation or down-regulation of carotenogenic genes associated with carotenoid biosynthesis pathway, and the differences in the expression of genes related to plastid synthesis, such as *PAP* gene, rather than the difference in the expression of a single gene. We inferred that differential expression patterns of carotenoids accumulation and plastid synthesis related genes have a strong influence on the carotenoids accumulation in loquat.
